# Efforts to Achieve Healthy Aging

**DOI:** 10.4137/imi.s976

**Published:** 2008-10-29

**Authors:** Ping-Chung Leung

**Affiliations:** Institute of Chinese Medicine, The Chinese University of Hong Kong, HKSAR

**Keywords:** chinese medicine, aging

## Abstract

Longevity is a blessing as long as good health is not lost. However, the tendency to have a decline on normal physiological activities is inevitable because of the natural processes of degeneration at all levels: molecular, cellular and organic. Hence, the elderly people frequently suffer from cardiovascular problems and skeletal deteriorations that gradually develop to disabilities. Awareness of factors leading to unhealthy aging has led to the formation of different professional groups that aim at the maintenance of health of aging community. The approach tends to be target orientated for the European and US groups, aiming at hormonal replacements and detoxification. In contrast, the oriental groups have been keeping their traditional belief of prevention and internal balance, using nutritional arrangements and non-strenuous exercise as means of maintaining health.

## Introduction

The twenty first century is characterized by the successes of modern science which have brought about apparently unlimited materialistic achievements. While people enjoy better health and remarkable means of healing which allow them to live to a much longer age, longevity and the increasing number of elderlies have produced new social problems (Buckwalther et al. 2003).

**Figure 1.**
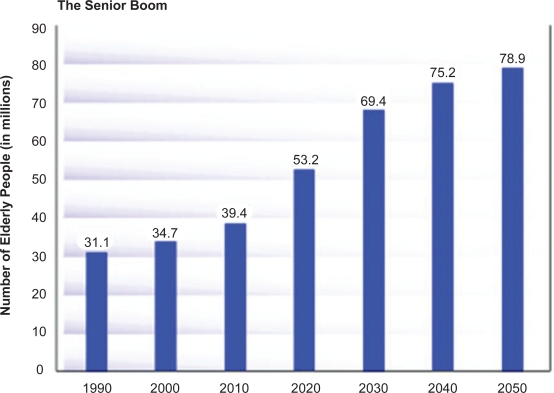
Senior Boom in U.S.A.

Figure 1 illustrate that the current expected age of survival for men and women all over the world is ever increasing, it has become clear that many of the surviving people with higher ages are in fact not in good shape, either socio-economically or physically (Lipsitz, 2004). In Hong Kong, for instance, the expected ages of survival have already increased to 79.3 for men and 85.4 for women, from the 2007 vital events by Census and Statistics Department. If these elderly citizens are in good health, they enjoy being senior and wise. On the contrary, if they are suffering from ill health, they become a burden to themselves, and their family and community needs to reserve huge resources to help them. Do we have healthy environments for the aging people to remain healthy?

The answer appears to be unfavorable. Firstly, the physically observable environment being presented to everyone is gloomy, particularly for Asia. The over-rapid economic growth in most regions have polluted the atmosphere, water sources, vegetations, living objects, the crops and the live-stocks. The direct effects could be the cause of general rise in the incidence of cancers. Economic growth has brought along socio-psychological tensions in the living environment of most inhabitants. The ever increasing stress has been rightly taken as the culprit of the ever declining mental health of all people, young and elderly alike.

The advances made in molecular biology has brought on the discovering and marking of the complete genomic picture, followed by the identification of specific genes responsible for rare, congenitally related disease entities. Optimists hurried to assume that sooner or later, all diseases and pathologies could be traced to a genomic origin and henceforth, be ultimately eliminated. Optimists have been predicting, that, individualized treatment according to different targets identified, would not be a remote practice. The different genomic make-up of different individuals, could certainly explain the commonly observed different responses to the similar treatment regime among patients labeled with the similar pathology. (Vaillancourt, 2002).

All appear well and another major medical advance in the direction of absolute core is expected, until again, further advances in molecular biology reveals much more complicated pictures. Pathologies and diseases, at molecular levels, i.e. beyond tissues, cellular and finer histomorphometric levels, viz, proteomic levels, are under the influence and control of very complicated systems. These systems interact and remain in an unstable equilibrium. Once the unstable equilibrium is disturbed, the individual will be bothered with pathological changes which may or may not be linked with symptoms and signs. The mechanisms affecting the different systems of proteomic reactions are influenced not only by the genomic make-up of the individual, but also by the environment the individual faces. The environment is both external and internal. The external environment has been discussed. The internal environment refers to the alimentary tract, the respiratory and excretory tracts, all in tubular forms of various shapes. The living organisms inside these tubular cavities are ever-changing and the personal habits, life-styles and individual activities are initiating environmental changes all the time. One might argue, whatever and whenever changes occur, counter-changes to bring stability and equilibrium are always possible. Be it true as it may, where is the concerted motivation and concerted dynamic which is expected to be essential for positive stability? (Vaillancourt, 2002). With aging, one expects much higher chances of losing the stability. Keeping healthy with aging is therefore, of utmost importance.

## Aging: Changes at Different Levels

Aging is a normal life process which involves a complexity of objective changes in the pathological deterioration of cells and tissues in the phenomenon know as degeneration. Normally cells undergo repair and regeneration in response to wearing out. With aging, their proliferative capacity declines, their differentiation into specialized functions slows down, and their normal responses to growth factors become hazardous. Laboratory explorations have suggested that these degenerative changes could have resulted from free radical accumulations or abnormal protein productions.

Cellular degenerations lead directly to tissue and organ damages, either directly through declines in circulation, loss of muscle and bone masses, or indirectly through inactive hormonal activities, inefficient immunological reactions, or loss of stem cell supplementation.

Among the widespread, generalized phenomena, which leave no cells, tissues or organs unaffected, there are areas, which are either more severely affected, or, when they are affected, would lead to readily expressed pathological changes, thus leading to immediate manifestations of health deterioration, followed shortly by the occurrence of diseases. The critical area include cardio-vascular degenerations as a result of abnormal cholesterol deposits leading to coronary and cerebral artery obstructions; abnormal cellular proliferations, leading to cancer development; musculoskeletal deteriorations leading to physical weakness and pain, and proneness to injuries. One area that is becoming more prominent in recent years because of the further increases in survival age, is related to the loss of neurological regenerative power, leading to a variety of dementia. Beyond the tissues and cells, lower down at the molecular levels, normally the proteomic activities maintain a balanced signaling which keeps generation and degeneration, anabolism and catabolism at a balance. Aging refers to a stage when deterioration becomes more rapid, thus giving manifestations of different degrees of frailty. Opposing couples of cell signaling are found (though not yet completely understood) in all the structural and function balance of the body tissues.

## Assessing Patient Needs and Providing Treatment

With the serious challenges from aging, there is a natural enthusiasm to positively respond to this urgent need of the new century. The enthusiasm comes not only from the elderlies themselves and the professional healers, but also from the Health Service providers on the Government level.

In the Affluent World, the market-driven economy has initiated the formation of special professional groups to provide services to the aging people. One remarkable blooming group has arisen from Europe—the Anti-aging Medicine Specialization (AAMS). Within a few years, this organization has been the most influential, first in Europe, then spreading to the United States. It holds frequent training courses for physicians and paramedicals. It facilitated the establishment of specific clinics that offer unique Healthy aging related advice, clinical investigations and treatment. The major areas of concern from the AAMS are related to Nutrition, Hormonal deficiencies, Psychological health, Environment and Physical Activities.

On the nutrition side: healthy food and healthy drinks are redefined. So are bad food and bad drinks, so that followers would not take milk products, sugar, unsprouted grains, alcoholic and caffeinated drinks (Werbach, 1993). A new concept on food digestion is established, which emphasizes on subclinical food allergy related to specific good items which initiate a major change of intestinal flora (Gibson, 2006). To prove the postulations, specific tests are created. “Toxicity” of food items is also in the lime-light. A new disease syndrome—“Chronic Fatigue Syndrome” has emerged, assuming that the cause is due to an imbalance produced by an insufficiency of the essential food substances and the excess of “toxic” chemicals. (Yoshimura, 1985; McKenna, 2006).

The Hormonal insufficiency school commands even greater respect since the scientific basis of the imbalance between opposing internal secretions appears a very sound theory. We are all used to “healthy eating” and “healthy drinking” with which consideration, food and drinks considered to be good for health are particularly stressed and listed for recommended consumption. To date, not only is healthy food and drink redefined, but other items are analyzed and labeled as being bad for health. Such “bad” items are not short of surprises, as for example, milk products, sugar, unsprouted grains, and alcoholic drinks, have been labeled as “bad” food. (Werbach, 2003) The rationale for such consideration is based on a new concept of food digestion which takes into consideration the possible occurrence of food allergy which could be sub-clinical. A food item traditionally taken as good, e.g. milk products, is likely to be the cause of sub-clinical allergy, therefore, could become bad for health. Such items induce allergy through a complicated mechanism of changing the intestinal flora (Gibson, 2006). “Toxicity” is redefined as not only a state that produces direct adverse effects on the normal physiology, but the accumulative results of sub-clinical allergy initiated through the consumption of “bad” food.

“Chronic fatigue syndrome” is a term commonly used for the aging people and the cause of this syndrome has been attributed to the careless consumption of food which suffers an imbalance between a sufficient amount of essential food items and an excess of “toxic” items.

An analysis of the “toxic” effects could be accomplished via a careful history taking on the nutritional intake. At the same time, enthusiasts have been creating new laboratory tests to confirm the existence of allergy and “toxicity”.

When serological tests are used to detect imbalances, titre values of opposing internal secretions are observed. Such are manifestations of hormonal imbalances.

Normally only gross imbalances are considered significant in clinical situations. Now that sophisticated tests to measure minute deficiencies are available to confirm small insufficiencies or overactivities, ailments like “chronic fatigue syndrome” and “subclinical allergy” that still lack perfect objective definitions appear to have reliable pathological explanations (Lindstedt, 2001; Krabbe, 2004). Before the establishment of such evidences, the aging people are advised to rely on exercises and a variety of harmless measures like meditations and spiritual involvements to help themselves. These measures could now be combined with a sophisticated hormonal control (either supplement or suppression), administrated through medical attention.

These current developments are really getting popular. However, if one applies principles of evidence-based medicine to critically evaluate the efficacy of the treatment, one might be disappointed because the parameters are yet to be properly defined.

There is yet one other important consideration in the attempt to uncover the black box of fatigue and aging. Pollution in the atmosphere and environmental water in the sea and rivers is deteriorating so rapidly that one has little reservation associating one’s physiological distress with pollution. Unclean air and contaminated water certainly would not do anything good for the degenerating tissues. In contrast to the mounting interests on the pursuance of deductive practices to maintain health in the deteriorating process of aging in the affluent world, the oriental concept of anti-aging has kept its popularity.

## The Oriental Concepts

The oriental concept of longevity emphasizes less on the external influences but concentrates more on an internal balance. The Yin-Yang forces need to be kept under harmonious balance, without which, the living individual suffers either an over- or under—active state. The balanced state relies on a Holistic equilibrium. The holistic equilibrium is mainly internal, but also needs to be harmonious with the environment, i.e. respecting climatic and geographic changes. One’s position in the family and community needs to be kept in harmony too, without which, one’s internal balance will be lost (Leung, 2001). The means and efforts to initiate a reasonable state of holistic equilibrium have not been medication orientated.

One of the essential requirements towards the achievement of the internal harmony is balanced nutrition. Balanced nutrition refers to a regular food consumption according to the need, a balanced diet which avoids the choice of rich entities, a careful selection of fresh and hygienic items and, sometimes, using herbal choices to help keeping a good balance (Liang, 2006). For the Chinese people, gourmet recipe often contain items of herbs that are commonly known as vegetables, although the same items might be medicinal choices for special uses. Indulgence on rich food and drink is strictly not recommended. Table 1 listed some of the vegetable herbs.

There are also specific food items which are better known for their use as anti-aging selections. Although some of these are leading items in classical formulations recommended for specific treatment, they could still be used as food. Table 2 listed some of the popular examples.

The oriental way of assessing patient needs among the aging population does not rely on specific symptoms, signs or laboratory tests. Actually when the manifestations of aging, be it chronic fatigue, different degrees of disability or frailty, appearance, it is considered too late. The practice of achieving the holistic balance needs to have proactive measures which could actively prevent the appearance, or premature appearance of the aging syndrome.

The oriental practice gives a special term to this active promotion of stable health, viz “Treating before falling ill”. The literal term refers to an active practice which includes physical activities, nutritional considerations and a peaceful mind. The literal term might apparently resemble disease prevention in the public health sense. However, this prevention and “treating before falling ill” refers to a personal level. So it is a personal practice to maintain personal health. With the modern concept of immunology, this practice might be understood as personal practices that bring the immuno-defense system to an efficient level which helps to maintain a good balanced healthy state. Apart from a healthy life-style, which includes the choice of favorable living environments, the oriental practice of health maintenance often actively include the consumption of special natural products, vegetables and fruits, all of which could be considered herbal medicinal items.

In recent years, a lot of interest has gathered around the demonstration of favorable immunological responses to the consumption of herbal items, and many positive evidences have been achieved. (Imazu, 2006; Miura, 2007) To quote just a few examples, fungal products, ganoderma (Wang, 1997; Guo, 2004), coriolus (Wong, 2004), and cordyceps (Chen, 1991) have all been demonstrated in the laboratory to be immuno-supportive. Some herbs, on the other hand, have been found to be effective in the control of allergic responses (Tan, 2004; Zhao, 2005a; Zhao, 2005b).

While exercises have been advocated to be universally beneficial to health, in Europe and the U.S. strenuous types of exercise like ball games and aerobics have been popular. In The Orient, mild bodily exercises with a lot of static trainings, in the form of Taichi, or Qigong in China, and Yoga in India, emphasise more on the promotion of internal well-being (Gibson, 2006). The Journal of American Geriatric Society reported in April 2007 the results a randomized controlled trial of 112 subjects, aged 59–86, on Taichi exercises against health education, using chicken pox vaccine as a stimulant to immunological responses. Those in the Taichi group enjoyed a 40% increase in their immunological responses (double that of controls) and an improved physical and mental state of health was also observed.

It is interesting to look at Japan which has been famous with many champion regions of longevity. The Nobuyoshi Hirose Centenarian Research, reported that the life style of the people living beyond 100 years of age in Okinawa, Japan, share the following criteria:

- Consuming a healthy diet with good vitamins;
- Plenty of physical exercises;
- Sleeping a lot and facing little stress;
- Living in quiet, peaceful surroundings with fresh air, fresh water, but no excessive sunlight; and
- Keeping active in the community (Kazuhiko, 2007).

## Recommendations for Healthy Aging

Aging is a normal life process. To make it a happy event, one has to understand the physiological changes in response to the structural deteriorations with age. While the European group insistent on a scientific deductive approach to give endless efforts to detect minor changes inside the body (hormonal alterations), which might be responsible for the deteriorations and then act accordingly by either supplement or elimination. However, the oriental group continues its holistic approach of maintaining the internal balance. Health is a complex issue. The individual as a living organism must have its own in-born unique integrity, which although is inconvertible, is under the constant influence of environmental and socio-psychological changes. The summation of inborn state and fresh unending interferences create a most complicated situation that modern science, as yet fails to explain. Without the genuine explanation and identification of the cause of problems, specific treatment is difficult. At this stage it might be wise to adopt the holistic approach which allows a free personal choice, relying on the knowledge on food, physical activities and psycho-social wellbeing to maintain a harmonious balance.

Relying on deductive science to stop aging and promote longevity sounds promising and is familiar to us who enjoy the fruits of modern science. However, the deductive approach, in the first place, is not yet mature, and secondly, depends again on medications that unavoidably exploit our external and internal environments. The Holistic approach, aiming at maintenance of internal harmony could be considered a special form of Natural Healing which has little interference on the environment of the individual, both external and internal. (Benno, 1986; Hooper, 2001; Abreu, 2005).

## Figures and Tables

**Table 1.** Nutritional items favored in Chinese medicine.

**Chinese name**	**Chinese herbs**
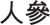	*Radix Ginseng*
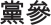	*Radix Codonopsis*
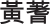	*Radix Astragali*
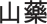	*Rhizoma Dioscoreae*
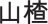	*Fructus Crataegi*
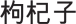	*Fructus Lycii*
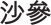	*Adenophora stricta Miq.*
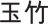	*Rhizoma Polygonati Odorati*
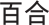	*Bulbus Lilii*
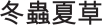	*Cordyceps*
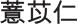	*Semen Coicis*
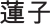	*Semen Nelumbinis*

**Table 2.** Anti-aging herbal items favored in Chinese medicine.

**Supplementing haemopoiesis:**	
**Chinese name**	**Chinese herbs**

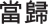	*Angrllica sinesis*
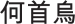	*Radix Polygoni Mulitiflori*
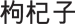	*Fructus Lycii*
**Supplementing Yin deficiency:**	
**Chinese name**	**Chinese herbs**
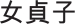	*Fructus Ligustri Lucidi*
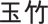	*Rhizoma Polygonati Odorati*
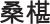	*Fructus Mori*
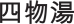	Siwutang
**Supplementing Yang deficiency:**	
**Chinese name**	**Chinese herbs**
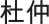	*Cortex Eucommiae*
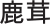	*Cornu Cervi Pantotrichum*
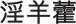	*Herba Epimedu*

The above herbs are generally used in food.
